# Longitudinal trajectories of burnout, perceived stress, and depressive symptoms among pediatric residents enrolled in professional master’s programs in China: a repeated-measures cohort study

**DOI:** 10.3389/fpubh.2026.1923629

**Published:** 2026-09-15

**Authors:** Dongdong You, Weikun Zheng, Hailin Zhang, Xiaohong Cai, Feng Li

**Affiliations:** 1 Department of Neonatology, The Second Affiliated Hospital and Yuying Children’s Hospital of Wenzhou Medical University, Wenzhou, China; 2 Department of Pediatric Infectious Diseases, The Second Affiliated Hospital and Yuying Children’s Hospital of Wenzhou Medical University, Wenzhou, China; 3 Department of Pediatric Respiratory Medicine, The Second Affiliated Hospital and Yuying Children’s Hospital of Wenzhou Medical University, Wenzhou, China; 4 Department of Pediatric Neurology, The Second Affiliated Hospital and Yuying Children’s Hospital of Wenzhou Medical University, Wenzhou, China

**Keywords:** burnout, depressive symptoms, emotional exhaustion, longitudinal study, pediatric residents, perceived stress, professional master’s programs, standardized residency training

## Abstract

**Background:**

Pediatric workforce sustainability is an important public health concern in China. Pediatric residents enrolled in professional master’s programs face simultaneous clinical training and academic demands, but longitudinal evidence on how their psychological burden changes during standardized residency training remains limited. This study aimed to characterize trajectories of burnout, perceived stress, and depressive symptoms across postgraduate year 1 (PGY1), postgraduate year 2 (PGY2), and postgraduate year 3 (PGY3).

**Methods:**

This longitudinal repeated-measures cohort study included 50 pediatric residents enrolled in professional master’s programs at a tertiary teaching hospital in China. Each resident was assessed once in PGY1, PGY2, and PGY3, yielding 150 repeated observations. Burnout was assessed using the Maslach Burnout Inventory–Human Services Survey, perceived stress using the 10-item Perceived Stress Scale, and depressive symptoms using the Patient Health Questionnaire-9. Generalized estimating equation models with robust standard errors were used to estimate stage-related mean differences while accounting for within-person clustering and adjusting for sex and cohort year. An exploratory participant-level analysis enumerated each participant’s categorical burnout status across PGY1–PGY3 using the two previously defined burnout indicators.

**Results:**

Across all repeated observations, mean scores were 27.65 ± 6.34 for emotional exhaustion, 15.80 ± 3.89 for depersonalization, 24.27 ± 5.48 for personal accomplishment, 67.19 ± 7.07 for the exploratory composite burnout index, 20.77 ± 5.87 for perceived stress, and 14.16 ± 4.72 for depressive symptoms. Descriptively, emotional exhaustion and the exploratory composite burnout index were higher at PGY3 than at PGY1. In adjusted GEE models, PGY3 was associated with higher emotional exhaustion compared with PGY1 (*β* = 2.740, 95% CI: 0.535–4.945, *p* = 0.0149) and a higher exploratory composite burnout index (*β* = 2.920, 95% CI: 0.417–5.423, *p* = 0.0222). No statistically significant PGY3-versus-PGY1 differences were observed for depersonalization, personal accomplishment, perceived stress, or depressive symptoms after adjustment. Participant-level transition analysis showed that 42 of 50 residents (84.0%) screened positive for burnout at all three stages, whereas 8 of 50 (16.0%) met the stricter three-dimension high-burnout definition at all three stages.

**Conclusion:**

Among pediatric residents enrolled in professional master’s programs, the clearest stage-related difference was higher emotional exhaustion at PGY3, with a parallel increase in the exploratory composite burnout index. This pattern is consistent with greater occupational exhaustion in later training, but causal attribution to training stage is not possible. Repeated, stage-specific monitoring and targeted residency support may help protect trainee wellbeing and strengthen the sustainability of the pediatric workforce in China.

## 1 Introduction

Pediatric workforce sustainability has become an important public health issue in China (1–3). Pediatricians have long faced heavy workloads, frequent doctor–patient communication challenges, high emotional demands, and a persistent shortage of qualified professionals (1–3). These pressures are not limited to senior physicians; they also affect residents who provide front-line pediatric care while still undergoing professional training. Occupational psychological distress during this period may influence not only individual wellbeing, but also training quality, professional development, career retention, patient safety, and the stability of the pediatric workforce (4–7). Burnout, perceived stress, and depressive symptoms among pediatric residents should therefore be understood as workforce and public health concerns rather than merely individual psychological problems.

Pediatric residents enrolled in professional master’s programs represent a particularly important group in this context. In China, these residents are required to complete a three-year standardized residency training program while also meeting academic and research requirements associated with professional degree training (8, 9). In the present study, the first, second, and third years of standardized residency training are referred to as postgraduate year 1 (PGY1), postgraduate year 2 (PGY2), and postgraduate year 3 (PGY3), respectively. These stages are not interchangeable categories. PGY1 is often characterized by adaptation to hospital workflow, supervised clinical practice, and role transition, whereas PGY2 and PGY3 may involve increasing clinical responsibility, academic pressure, qualification assessment, research output, employment concerns, and professional identity formation. Thus, pediatric residency is best viewed as a time-ordered occupational exposure in which psychological burden may change as training progresses.

Existing research has provided important evidence on the mental health burden of pediatric residents, but most available studies have been cross-sectional (10–12). A closely related study published in Frontiers in Public Health reported substantial levels of stress, anxiety, and depression among Chinese pediatric residents and identified meaningful associations among social support, psychological resilience, burnout, and negative emotions (10). However, cross-sectional studies cannot determine whether psychological burden worsens, improves, or fluctuates within the same residents as they move from PGY1 to PGY3. More broadly, comparing different trainees at a single time point can help identify who appears vulnerable, but it cannot adequately answer when risk increases or which training stages may require targeted screening and support. Longitudinal evidence following the same pediatric residents across the three training years is therefore needed to inform stage-specific residency management and mental health intervention.

Burnout, perceived stress, and depressive symptoms are related but distinct outcomes that should be assessed together. Emotional exhaustion and depersonalization reflect occupational depletion and disengagement, while personal accomplishment captures the professional efficacy dimension of burnout (13). Perceived stress reflects residents’ appraisal of pressure and coping capacity (14), whereas depressive symptoms indicate broader affective and cognitive distress (15). These constructs may overlap during pediatric training because residents are simultaneously exposed to clinical uncertainty, emotional labor, academic expectations, and professional evaluation. However, their trajectories may not be identical. Burnout may be more sensitive to cumulative workplace exposure, whereas perceived stress and depressive symptoms may also be influenced by broader psychosocial and personal factors. A repeated-measures framework allows these outcomes to be examined in parallel and helps clarify whether psychological burden during pediatric residency is static, stage-dependent, or progressively accumulating.

Accordingly, the present study aimed to characterize longitudinal changes in burnout, perceived stress, and depressive symptoms among pediatric residents enrolled in professional master’s programs and undergoing standardized residency training in China. Five enrollment cohorts from 2018 to 2022 were followed, and each participant was assessed once during PGY1, PGY2, and PGY3, yielding 150 repeated observations from 50 residents. Burnout was assessed using the Maslach Burnout Inventory–Human Services Survey, including emotional exhaustion, depersonalization, and personal accomplishment, together with a composite burnout index (13). Perceived stress was measured using the 10-item Perceived Stress Scale (14), and depressive symptoms were assessed using the Patient Health Questionnaire-9 (15). Because repeated measurements from the same resident are correlated and should not be treated as independent observations, generalized estimating equations were used to evaluate stage-related differences while accounting for within-person clustering and adjusting for sex and cohort year (16). We hypothesized that psychological burden would vary across training stages, with the clearest stage-related change expected in burnout-related outcomes as residents advanced through training. By focusing on within-cohort longitudinal change rather than cross-sectional comparison between different trainee groups, this study seeks to provide evidence relevant to public mental health, stage-specific residency support, and the maintenance of a stable and well-supported pediatric workforce in China.

## 2 Methods

### 2.1 Study design and participants

This longitudinal repeated-measures cohort study was conducted among pediatric residents enrolled in professional master’s programs and undergoing standardized residency training at a tertiary teaching hospital in China (8). Five enrollment cohorts entering training between 2018 and 2022 were followed across the 3 years of standardized residency training. In this study, the first, second, and third years of standardized residency training were defined as postgraduate year 1 (PGY1), postgraduate year 2 (PGY2), and postgraduate year 3 (PGY3), respectively. Each resident was assessed once during each training stage, generating a balanced repeated-measures panel with three observations per participant.

Eligible participants were pediatric residents enrolled in professional master’s programs who were undergoing standardized residency training. At recruitment, 50 residents met these eligibility criteria; all 50 provided written informed consent and were enrolled (participation rate 100% among the identified eligible residents). No participant was excluded after enrollment for incomplete questionnaire data, and all 50 completed the scheduled PGY1, PGY2, and PGY3 assessments. Thus, there was no loss to follow-up within the enrolled cohort, and the final analytic dataset comprised 50 participants and 150 repeated observations. The participant-selection and longitudinal follow-up flow is shown in Figure 1, and participant-level and observation-level characteristics are summarized in Table 1.

**Figure 1:**
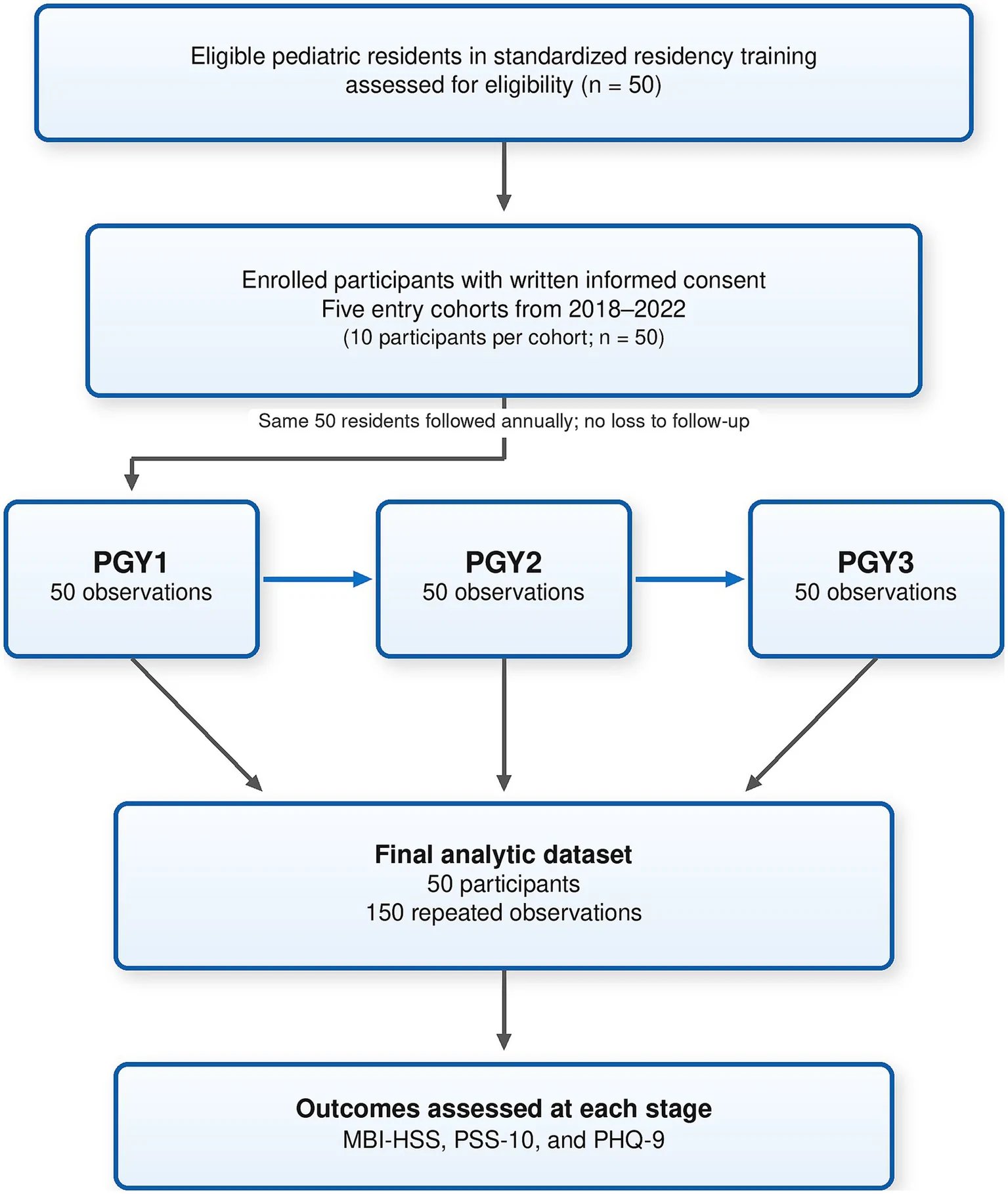
Participant selection and longitudinal follow-up flow. Arrows from PGY1 to PGY2 and from PGY2 to PGY3 indicate continuity of the same 50 participants across the three annual assessments.

**Table 1:** Study design and participant characteristics of the longitudinal cohort.

Characteristic	Category	Participants, *n* (%)	Repeated observations, *n* (%)
Total	–	50 (100.0)	150 (100.0)
Sex	Male	25 (50.0)	75 (50.0)
	Female	25 (50.0)	75 (50.0)
Enrollment cohort	2018	10 (20.0)	30 (20.0)
	2019	10 (20.0)	30 (20.0)
	2020	10 (20.0)	30 (20.0)
	2021	10 (20.0)	30 (20.0)
	2022	10 (20.0)	30 (20.0)
Training stage	PGY1	–	50 (33.3)
	PGY2	–	50 (33.3)
	PGY3	–	50 (33.3)
Observation period	2018–2024	–	150 (100.0)
Repeated measurement completion	3 records per participant	50 (100.0)	150 (100.0)

PGY, postgraduate year. Participants are unique residents; repeated observations are clustered within participant ID in the GEE analysis.

Fifty eligible pediatric residents enrolled in professional master’s programs were identified across five entry cohorts from 2018 to 2022 (10 participants per cohort). All 50 provided written informed consent and were enrolled; no eligible resident declined participation after eligibility was established. The same 50 participants completed one assessment at postgraduate year 1 (PGY1), postgraduate year 2 (PGY2), and postgraduate year 3 (PGY3), as shown by the sequential arrows. No participant was lost to follow-up or excluded after enrollment for incomplete repeated measurements. The final analytic dataset therefore contained 50 participants and 150 repeated observations. Psychological outcomes were assessed using the MBI-HSS, PSS-10, and PHQ-9.

The study received ethical approval from the hospital’s ethics committee (2021-K-167-02). Written informed consent was obtained from all participants before data collection. Participation was voluntary, and all data were anonymized using participant identification codes.

### 2.2 Data collection procedure

Data were collected during prespecified annual assessment periods across the three standardized residency training years. Participants completed self-administered questionnaires assessing occupational burnout, perceived stress, and depressive symptoms. To ensure longitudinal linkage while protecting confidentiality, each resident was assigned a unique participant identification code. The same code was used to link that resident’s PGY1, PGY2, and PGY3 assessments. The analytic dataset was structured in long format, with one row representing one participant’s observation at one training stage. Participant ID was used to identify repeated observations from the same resident, and training stage was specified as the primary time-ordered exposure.

### 2.3 Measures

#### 2.3.1 Sociodemographic and training-related variables

Participant-level variables included sex and enrollment cohort. Sex was treated as a time-invariant covariate. Cohort year referred to the year in which the resident entered standardized residency training and ranged from 2018 to 2022. Training stage was coded as PGY1, PGY2, and PGY3 and was prespecified as the primary exposure variable.

#### 2.3.2 Maslach Burnout Inventory–Human Services Survey

Burnout was assessed using the Maslach Burnout Inventory–Human Services Survey (MBI-HSS), a 22-item instrument comprising three subscales: emotional exhaustion (EE, 9 items), depersonalization (DP, 5 items), and personal accomplishment (PA, 8 items) (13). Each item is rated on a 7-point Likert scale from 0 to 6. Higher EE and DP scores indicate greater burnout burden, whereas higher PA scores indicate greater personal accomplishment and therefore lower burnout.

In the primary burnout analyses, EE, DP, and PA were analyzed separately in their original directions. In addition, an exploratory composite burnout index was calculated as EE + DP + (48 − PA), where 48 is the maximum possible PA score. Reversing PA placed all three dimensions in the same risk direction, so that higher values represented greater overall burnout burden and provided a compact descriptive summary across the three dimensions. This formula is not an official MBI-HSS scoring method and was not assumed to represent a validated unidimensional burnout construct; accordingly, the three validated MBI-HSS subscales remained the principal burnout outcomes, and the composite index was interpreted only as supplementary and exploratory. It was not used for diagnostic classification or clinical decision-making. For supplementary descriptive analyses, burnout screening positivity was defined as EE ≥ 27 or DP ≥ 10, and high burnout by all three dimensions was defined as EE ≥ 27, DP ≥ 10, and PA ≤ 33 (13). These cutoffs were used as descriptive screening indicators rather than diagnostic definitions. Internal consistency was evaluated descriptively. Cronbach’s alpha was 0.734 for EE, 0.584 for DP, and 0.631 for PA. The alpha for all 22 MBI-HSS items was 0.843; however, because the instrument is multidimensional, this value was not interpreted as evidence for a unidimensional total scale.

#### 2.3.3 Perceived Stress Scale-10

Perceived stress was assessed using the 10-item Perceived Stress Scale (PSS-10) (14). Each item is rated from 0 to 4, yielding a total score range of 0–40, with higher scores indicating greater perceived stress. Items 4, 5, 7, and 8 were reverse-scored before all 10 items were summed to calculate the PSS-10 total score (14). For descriptive severity classification, PSS-10 scores were categorized as low (0–13), moderate (14–26), and high (27–40). In the present study, the PSS-10 showed good internal consistency (Cronbach’s *α* = 0.814).

#### 2.3.4 Patient Health Questionnaire-9

Depressive symptoms were assessed using the Patient Health Questionnaire-9 (PHQ-9), a 9-item self-report instrument scored from 0 to 3 per item, with a total score range of 0–27 (15). Higher scores indicate more severe depressive symptoms. For descriptive severity classification, PHQ-9 scores were categorized as none/minimal (0–4), mild (5–9), moderate (10–14), moderately severe (15–19), and severe (20–27) (15). For supplementary descriptive analyses, PHQ-9 screening positivity was defined as PHQ-9 ≥ 10, and moderately severe or severe symptom levels were defined as PHQ-9 ≥ 15. These categories indicate symptom severity and do not establish a clinical diagnosis. In the present study, the PHQ-9 demonstrated good internal consistency (Cronbach’s *α* = 0.836).

### 2.4 Outcome definitions

The primary continuous outcomes were MBI-EE, MBI-DP, MBI-PA, the exploratory composite burnout index, PSS-10 total score, and PHQ-9 total score. The main exposure was training stage, defined as PGY1, PGY2, and PGY3. Sex and cohort year were included as prespecified covariates. Descriptive categorical outcomes included PSS-10 severity category, PHQ-9 severity category, burnout screening positivity, high burnout by all three dimensions, PHQ-9 ≥ 10, and PHQ-9 ≥ 15. These categorical variables were interpreted as screening classifications and were used for descriptive and supplementary analyses rather than as primary inferential outcomes.

### 2.5 Statistical analysis

Descriptive statistics were used to summarize participant characteristics and outcome distributions. Continuous variables are presented as mean ± standard deviation (SD), and categorical variables as counts and percentages. Participant-level characteristics and repeated-measurement structure are presented in Table 1. Stage-specific and sex-stratified descriptive summaries of scale scores are shown in Table 2. Severity distributions and screening indicators for perceived stress, depressive symptoms, and burnout-related classifications are presented in Supplementary Table S1.

**Table 2:** Descriptive scale scores by training stage and sex.

Outcome	Overall (*N* = 150)	PGY1 (*n* = 50)	PGY2 (*n* = 50)	PGY3 (*n* = 50)	Observed change: PGY3–PGY1	Male (*n* = 75)	Female (*n* = 75)
Burnout-related outcomes							
MBI-EE	27.65 ± 6.34	26.50 ± 6.22	27.22 ± 6.71	29.24 ± 5.87	+2.74	26.91 ± 5.72	28.40 ± 6.87
MBI-DP	15.80 ± 3.89	15.20 ± 4.15	15.76 ± 3.57	16.44 ± 3.91	+1.24	15.64 ± 3.87	15.96 ± 3.93
MBI-PA	24.27 ± 5.48	23.92 ± 5.96	23.90 ± 5.11	24.98 ± 5.38	+1.06	23.88 ± 5.36	24.65 ± 5.61
MBI burnout index	67.19 ± 7.07	65.78 ± 7.67	67.08 ± 6.92	68.70 ± 6.39	+2.92	66.67 ± 6.60	67.71 ± 7.52
Perceived stress							
PSS-10 total	20.77 ± 5.87	20.50 ± 6.40	20.18 ± 5.29	21.62 ± 5.90	+1.12	20.51 ± 5.80	21.03 ± 5.97
Depressive symptoms							
PHQ-9 total	14.16 ± 4.72	13.50 ± 5.01	13.80 ± 4.18	15.18 ± 4.86	+1.68	13.95 ± 4.59	14.37 ± 4.88

Values are presented as mean ± SD. The observed change represents the unadjusted descriptive difference between PGY3 and PGY1. Formal longitudinal inference was based on generalized estimating equation models, as shown in Table 3.

The primary longitudinal analyses were conducted using generalized estimating equation (GEE) models for repeated measurements (16). The analytic unit was the repeated observation, and repeated observations were clustered within participant ID to account for within-person correlation across PGY1–PGY3. Separate GEE models were fitted for MBI-EE, MBI-DP, MBI-PA, the exploratory composite burnout index, PSS-10 total score, and PHQ-9 total score. Because these outcomes were analyzed as continuous scores, models were specified with a Gaussian distribution and identity link function. An exchangeable working correlation structure was selected as a parsimonious specification for the three equally spaced repeated assessments, because it requires estimation of a single within-person correlation parameter and avoids the additional parameters required by an unstructured correlation matrix in a cohort of 50 participants. Robust sandwich standard errors were used so that inference was less dependent on exact specification of the working correlation structure. No formal sensitivity analysis comparing alternative working correlation structures was performed, and this is acknowledged as a methodological limitation.

Training stage was entered as a categorical predictor, with PGY1 as the reference category. Sex was included as a covariate, with male residents as the reference group. Cohort year was modeled as a continuous covariate representing a 1-year increase in the year of entry into standardized residency training. Regression coefficients are reported as adjusted mean differences with robust standard errors, 95% confidence intervals (CIs), and two-sided *p* values. A two-sided *p* value <0.05 was considered nominally statistically significant. Because six related but conceptually distinct continuous outcomes were evaluated in a modest single-center sample, no formal multiplicity adjustment was applied. The *p* values should therefore be interpreted as nominal rather than family-wise error-controlled results. Interpretation emphasized effect estimates and 95% CIs, and findings for the composite burnout index and supplementary analyses were treated as exploratory and hypothesis-generating.

As an exploratory supplementary analysis, Pearson correlation coefficients among key continuous outcomes, including MBI-EE, MBI-DP, the composite burnout index, PSS-10, and PHQ-9, were calculated using all 150 repeated observations and visualized as a correlation heatmap in Supplementary Figure S1. Because repeated observations from the same participants are not statistically independent, these correlations were interpreted as descriptive co-variation rather than causal or longitudinal associations.

As an exploratory secondary analysis, a participant-level transition analysis was conducted using the two previously defined categorical burnout indicators. Burnout screening positivity was defined as EE ≥ 27 or DP ≥ 10, and high burnout across all three dimensions was defined as EE ≥ 27, DP ≥ 10, and PA ≤ 33. For each definition, every participant’s PGY1–PGY3 status sequence was enumerated, and persistent positivity, PGY1-positive/PGY3-negative, and PGY1-negative/PGY3-positive patterns were summarized as counts and percentages. Because this secondary analysis was descriptive and dependent on cut points, no additional hypothesis testing was performed.

No missing values were observed for variables included in the final repeated-measures analytic dataset, and no imputation was therefore performed. Statistical analyses were conducted using R version 4.4.2 (R Foundation for Statistical Computing, Vienna, Austria) (17). Generalized estimating equation models were fitted using the geepack package (18), internal consistency reliability was assessed using the psych package (19), and figures were generated using the ggplot2 package (20).

## 3 Results

### 3.1 Participant characteristics and repeated-measurement structure

A total of 50 pediatric residents enrolled in professional master’s programs were included in the final longitudinal cohort. Each resident completed assessments at PGY1, PGY2, and PGY3, yielding 150 repeated observations for analysis. The cohort included 25 male and 25 female residents. Enrollment was evenly distributed across the five entry cohorts from 2018 to 2022, with 10 residents in each cohort. Each training stage contributed 50 observations. The study design and repeated-measurement structure are shown in Figure 1, and participant-level and observation-level characteristics are summarized in Table 1.

### 3.2 Descriptive trajectories of burnout, perceived stress, and depressive symptoms

Across all repeated observations, the mean scores were 27.65 ± 6.34 for emotional exhaustion, 15.80 ± 3.89 for depersonalization, 24.27 ± 5.48 for personal accomplishment, 67.19 ± 7.07 for the composite burnout index, 20.77 ± 5.87 for PSS-10, and 14.16 ± 4.72 for PHQ-9. Detailed descriptive statistics by training stage and sex are shown in Table 2.

Emotional exhaustion and the exploratory composite burnout index were higher at PGY3 than at PGY1 (29.24 vs. 26.50 and 68.70 vs. 65.78, respectively). The corresponding unadjusted PGY3-minus-PGY1 differences were +1.24 for depersonalization, +1.06 for personal accomplishment, +1.12 for PSS-10, and +1.68 for PHQ-9. These values are descriptive only; formal inference regarding stage-related differences was based on the adjusted GEE models in Table 3.

**Table 3:** Longitudinal generalized estimating equation models for repeated measurements.

Outcome domain	Outcome	Predictor	*β*	Robust SE	95% CI	*p* value
Burnout-related outcomes						
	MBI-EE	PGY2 vs. PGY1	0.720	1.136	−1.507 to 2.947	0.5263
		PGY3 vs. PGY1	2.740	1.125	0.535–4.945	0.0149^*^
		Female vs. Male	1.493	1.142	−0.744 to 3.731	0.1908
		Cohort year, per 1-year increase	1.153	0.446	0.280–2.027	0.0096^**^
	MBI-DP	PGY2 vs. PGY1	0.560	0.637	−0.688 to 1.808	0.3793
		PGY3 vs. PGY1	1.240	0.761	−0.251 to 2.731	0.1031
		Female vs. Male	0.320	0.767	−1.183 to 1.823	0.6764
		Cohort year, per 1-year increase	0.363	0.304	−0.233 to 0.959	0.2322
	MBI-PA	PGY2 vs. PGY1	−0.020	1.130	−2.234 to 2.194	0.9859
		PGY3 vs. PGY1	1.060	1.114	−1.123 to 3.243	0.3412
		Female vs. Male	0.773	0.869	−0.930 to 2.477	0.3737
		Cohort year, per 1-year increase	0.793	0.360	0.088–1.499	0.0275^*^
	MBI burnout index	PGY2 vs. PGY1	1.300	1.324	−1.295 to 3.895	0.3262
		PGY3 vs. PGY1	2.920	1.277	0.417–5.423	0.0222^*^
		Female vs. Male	1.040	1.283	−1.474 to 3.554	0.4175
		Cohort year, per 1-year increase	0.723	0.465	−0.188 to 1.635	0.1199
Perceived stress						
	PSS-10 total	PGY2 vs. PGY1	−0.320	1.117	−2.510 to 1.870	0.7746
		PGY3 vs. PGY1	1.120	1.200	−1.232 to 3.472	0.3506
		Female vs. Male	0.520	0.997	−1.433 to 2.473	0.6018
		Cohort year, per 1-year increase	1.057	0.403	0.268–1.846	0.0087^**^
Depressive symptoms						
	PHQ-9 total	PGY2 vs. PGY1	0.300	0.778	−1.226 to 1.826	0.6999
		PGY3 vs. PGY1	1.680	0.899	−0.082 to 3.442	0.0617
		Female vs. Male	0.427	0.900	−1.336 to 2.190	0.6353
		Cohort year, per 1-year increase	0.657	0.367	−0.062 to 1.375	0.0733

GEE, generalized estimating equation; *β*, adjusted mean difference; SE, standard error; CI, confidence interval. Models were adjusted for training stage, sex, and cohort year; participant ID was specified as the clustering variable to account for within-person correlation across repeated measurements. PGY1 and male sex were used as reference categories. Cohort year was modeled as a continuous covariate representing a 1-year increase in the year of entry into standardized residency training. ^*^p < 0.05; ^**^p < 0.01. MBI-PA should be interpreted in the original direction of personal accomplishment. The burnout index was used as an exploratory composite index and does not replace the three MBI-HSS dimensions. No multiplicity adjustment was applied; the composite burnout index and supplementary analyses were exploratory.

Panel A shows the mean exploratory composite burnout index across PGY1, PGY2, and PGY3. Panel B shows the mean PSS-10 score across training stages. Panel C shows the mean PHQ-9 score across training stages. Points and numeric labels indicate mean scores at each training stage, and shaded bands represent 95% confidence intervals around the mean. Each training stage included 50 repeated observations from the same 50 residents. Figure 2 displays group-level stage-specific means and confidence intervals rather than individual participant trajectories; stage-specific standard deviations are provided in Table 2. The figure is descriptive and should be interpreted together with the adjusted generalized estimating equation models reported in Table 3. PGY, postgraduate year; PSS-10, 10-item Perceived Stress Scale; PHQ-9, Patient Health Questionnaire-9.

**Figure 2:**
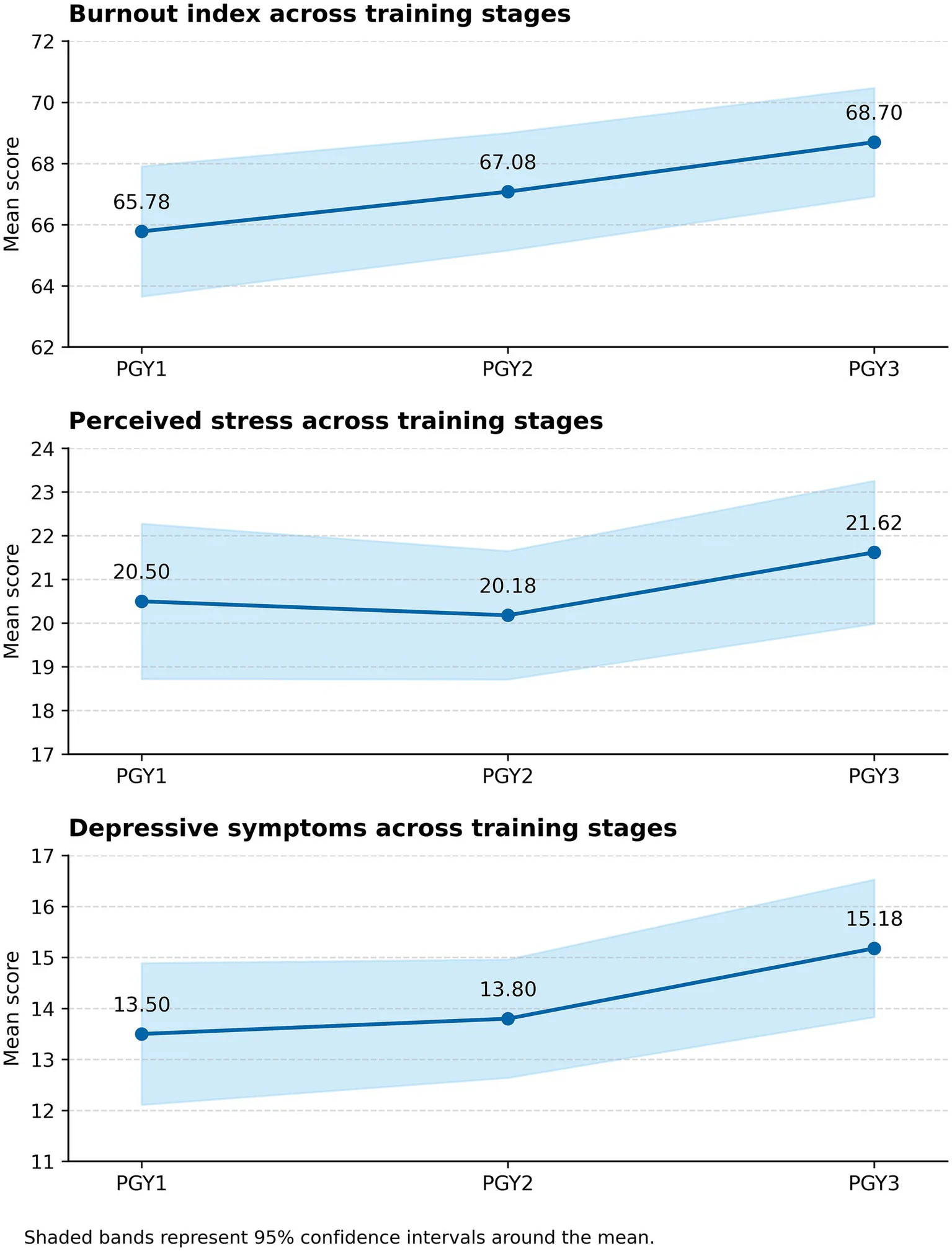
Longitudinal trajectories of burnout, perceived stress, and depressive symptoms across training stages.

Sex-stratified descriptive summaries showed slightly higher mean scores among female residents for most outcomes, but these differences were small and were further evaluated in the adjusted models.

### 3.3 Longitudinal GEE analyses

The primary longitudinal analyses were conducted using generalized estimating equation models adjusted for sex and cohort year, with participant ID specified as the clustering variable. The full model estimates are presented in Table 3.

Compared with PGY1, PGY2 was not significantly associated with any of the six continuous psychological outcomes. In contrast, PGY3 was significantly associated with higher emotional exhaustion compared with PGY1 (*β* = 2.740, robust SE = 1.125, 95% CI: 0.535 to 4.945, *p* = 0.0149). PGY3 was also associated with a higher exploratory composite burnout index (*β* = 2.920, robust SE = 1.277, 95% CI: 0.417 to 5.423, *p* = 0.0222). The clearest evidence of a stage-related difference was therefore observed for emotional exhaustion; the composite-index finding should be interpreted as supportive and exploratory.

No statistically significant PGY3-versus-PGY1 differences were observed for depersonalization, personal accomplishment, PSS-10, or PHQ-9 after adjustment. For PHQ-9 specifically, the adjusted PGY3-versus-PGY1 mean difference was 1.680 points (95% CI: −0.082 to 3.442, *p* = 0.0617); therefore, the numerically higher PGY3 mean should be regarded as a descriptive difference rather than statistically supported evidence of longitudinal worsening. The corresponding confidence intervals for depersonalization, personal accomplishment, and PSS-10 also included zero. Sex was not significantly associated with any primary continuous outcome in the adjusted models.

Cohort year showed significant positive associations with emotional exhaustion (*β* = 1.153 per year, robust SE = 0.446, 95% CI: 0.280–2.027, *p* = 0.0096), personal accomplishment (*β* = 0.793 per year, robust SE = 0.360, 95% CI: 0.088–1.499, *p* = 0.0275), and perceived stress (*β* = 1.057 per year, robust SE = 0.403, 95% CI: 0.268–1.846, *p* = 0.0087). Cohort year was not significantly associated with depersonalization, the composite burnout index, or PHQ-9 total score. These coefficients compare residents entering training in different calendar years and are distinct from the within-person PGY stage contrasts. Because cohort year was included as an adjustment variable rather than as the primary exposure, the estimates should not be interpreted as causal secular trends.

### 3.4 Supplementary severity distributions, reliability, and correlations

Supplementary Table S1 presents the categorical severity distributions for perceived stress, depressive symptoms, and burnout indicators. High perceived stress, defined as PSS-10 scores of 27–40, was observed in 27 of 150 repeated observations (18.0%) overall and was most frequent at PGY3. PHQ-9 screening positivity, defined as PHQ-9 ≥ 10, was observed in 120 of 150 repeated observations (80.0%). Moderately severe or severe symptom levels, defined as PHQ-9 ≥ 15, were observed in 66 of 150 repeated observations (44.0%) and were also most frequent at PGY3. These percentages describe screening categories and should not be interpreted as clinical diagnoses.

Burnout screening positivity, defined as emotional exhaustion ≥27 or depersonalization ≥10, was observed in 141 of 150 repeated observations (94.0%). High burnout by all three dimensions, defined as emotional exhaustion ≥27, depersonalization ≥10, and personal accomplishment ≤33, was observed in 82 of 150 repeated observations (54.7%) and increased descriptively from PGY1 to PGY3.

Internal consistency reliability estimates are shown in Supplementary Table S2. The PSS-10 and PHQ-9 showed good internal consistency, and emotional exhaustion showed acceptable internal consistency. Internal consistency was lower for depersonalization and personal accomplishment. The alpha for the full 22-item MBI-HSS item set was reported descriptively but was not interpreted as evidence of a unidimensional total scale.

Exploratory correlation analyses among key continuous outcomes are shown in Supplementary Figure S1. The strongest positive correlation was observed between PSS-10 and PHQ-9 (*r* = 0.801). Emotional exhaustion was positively correlated with depersonalization, the composite burnout index, perceived stress, and depressive symptoms. Because these correlations were calculated using repeated observations, they should be interpreted as descriptive co-variation rather than causal or longitudinal associations.

### 3.5 Exploratory participant-level burnout transitions

Using the screening definition of EE ≥ 27 or DP ≥ 10, 42 of 50 residents (84.0%) screened positive at PGY1, PGY2, and PGY3. Among the 45 residents who screened positive at PGY1, 2 (4.4%; 4.0% of the full cohort) screened negative at PGY3. All 5 residents who screened negative at PGY1 screened positive at PGY3 (100.0% of the baseline-negative group; 10.0% of the full cohort).

Using the stricter definition requiring high EE, high DP, and low PA simultaneously, 8 of 50 residents (16.0%) met the high-burnout criterion at all three stages. Of 21 residents meeting this definition at PGY1, 7 (33.3%; 14.0% of the full cohort) no longer met it at PGY3; conversely, 19 of 29 residents not meeting it at PGY1 (65.5%; 38.0% of the full cohort) met it at PGY3. The complete PGY1–PGY3 status sequences and conditional transition denominators are provided in Supplementary Table S3.

## 4 Discussion

This longitudinal repeated-measures study examined burnout, perceived stress, and depressive symptoms among pediatric residents enrolled in professional master’s programs during standardized residency training in China. The clearest stage-related finding was higher emotional exhaustion at PGY3 than at PGY1. The exploratory composite burnout index showed a parallel difference, but this supplementary measure is not an official MBI-HSS score and does not replace interpretation of the three subscales. In contrast, depersonalization, personal accomplishment, perceived stress, and depressive symptoms did not show statistically significant PGY3-versus-PGY1 differences after adjustment. These results are consistent with, but do not establish, greater occupational exhaustion in later training and should not be interpreted as uniform worsening across all psychological domains.

The magnitude of the emotional-exhaustion finding warrants cautious interpretation. The adjusted PGY3-versus-PGY1 mean difference was 2.74 points. Relative to the PGY1 standard deviation of 6.22, this corresponds to approximately 0.44 SD as a rough contextual measure of magnitude; it is not a formally standardized within-person effect size. The present study did not prespecify a minimum clinically important difference for MBI-EE, and statistical significance should therefore not be equated with clinically meaningful deterioration. Direct comparison with prior pediatric-residency studies is also limited because published studies have used heterogeneous burnout definitions, instruments, thresholds, and designs (7, 11, 12, 21). Emotional exhaustion represents the energy-depletion component of burnout and is plausibly related to prolonged occupational demands (22). Residents may face clinical workload, night duty, emotional labor, increasing responsibility, academic requirements, qualification assessment, employment concerns, and career planning. Prior evidence links long working hours and healthcare occupational demands with burnout burden (21, 23, 24, 34–36). However, these exposures were not measured in the present study, so they remain possible explanations rather than demonstrated mechanisms. The PA estimate was not statistically significant; accordingly, no inference about stage-related professional growth or decline in personal accomplishment is warranted from these data.

The present findings should not be interpreted as suggesting that perceived stress or depressive symptoms were unimportant. Descriptive analyses indicated substantial psychological symptom burden across training, but descriptive patterns must be distinguished from adjusted longitudinal inference. PHQ-9 scores were numerically higher at PGY3, yet the adjusted PGY3-versus-PGY1 difference did not reach conventional statistical significance (*β* = 1.680, 95% CI: −0.082 to 3.442, *p* = 0.0617). Thus, the data do not provide statistically significant evidence of a stage-related increase in depressive symptoms. PHQ-9 screening positivity and burnout screening indicators were common across the three stages, but these categories are screening classifications rather than clinical diagnoses. Previous meta-analytic evidence has shown that depressive symptoms are common among resident physicians (5), and depression and burnout have been associated with medical errors in physicians and pediatric residents (6, 7). The non-significant PSS-10 and PHQ-9 stage estimates may reflect limited precision, high symptom levels throughout training, or the influence of occupational and non-occupational factors; these possibilities cannot be distinguished with the present data.

These results extend previous research on pediatric residents and physician mental health. A closely related study of Chinese pediatric residents found that negative emotions were common and were associated with social support, psychological resilience, and burnout, but its cross-sectional design could not characterize within-person change across training (10). Studies from other pediatric residency settings have also suggested that burnout is related to the educational climate and training environment (11). More broadly, physician burnout research has shown substantial heterogeneity in definitions, instruments, and thresholds, which makes direct comparison of prevalence estimates difficult across studies and settings (12). Broader residency research has also shown that burnout is common among residents and may vary across specialty and training contexts (21). The present study adds within-person repeated measurements across PGY1, PGY2, and PGY3, while remaining limited to a single program. Cohort-year coefficients require a different interpretation from PGY stage effects: they contrast different entry cohorts rather than changes within the same resident. The positive cohort-year associations with EE, PA, and PSS-10 may reflect calendar-period influences, institutional changes, selection or composition differences between cohorts, or other unmeasured secular factors. Because these potential explanations were not measured and the directions were not uniform across outcomes, the cohort-year coefficients should be viewed as adjustment terms rather than evidence of a simple temporal worsening trend.

The participant-level findings add an important distinction to the stage-level averages. Screening positivity was highly persistent, but the stricter three-dimension classification was more dynamic: some residents recovered from a high-burnout classification while a larger group newly met it by PGY3. Thus, relatively stable or gradually increasing group means can coexist with clinically relevant movement between categorical states at the individual level. These cut-point-based transitions are descriptive and should not be interpreted as diagnostic incidence or remission.

Comparable evidence across the medical career span suggests that distress is already present before residency and remains important after training, although direct numerical comparisons are constrained by differences in instruments and definitions (Supplementary Table S4). In a national US study using validated single items adapted from the MBI, burnout was reported in 55.9% of medical students, 60.3% of residents/fellows, and 51.4% of early-career physicians (25). Among US pediatric residents assessed with the full MBI, prevalence was 56% in 2016 and 54% in 2017 (26). A Saudi pediatric residency study using the MBI-HSS reported high EE in 63.6%, high DP in 27.7%, low PA in 48.5%, and simultaneous high burnout across all three dimensions in 15.9% (27).

For the post-training context, a national Chinese survey of 25,120 medical professionals using the MBI-HSS reported high EE in 35.5%, high DP in 30.0%, low PA in 38.4%, and all three adverse dimensions in 11.2%; shorter work experience and pediatric practice were associated with higher burnout risk (28). In an Argentine pediatric hospital sample that included residents and staff pediatricians, mean EE, DP, and PA scores were 30.60, 10.51, and 35.76, respectively; longer pediatric practice was associated with lower DP and higher PA (29). Together, these studies do not establish a single universal trajectory, but they place the present PGY1–PGY3 pattern within a broader career-span narrative: burnout risk is evident before and during residency, while some domains—particularly depersonalization and professional accomplishment—may improve with experience even when emotional exhaustion remains substantial.

Given the single-center design and modest sample, the practical implications of these findings should be considered provisional and should be confirmed in larger multicenter studies. Residency programs may nevertheless consider repeated wellbeing assessment across training rather than relying only on an initial PGY1 assessment. If later-stage emotional exhaustion is replicated, potential responses could include confidential counseling and mentoring as well as organizational measures addressing workload distribution, night-shift burden, protected academic time, rotation design, supervisory support, and timely feedback. Individual-focused support should not substitute for structural improvement. Evidence from physician-burnout intervention research suggests that both individual-focused and organization-directed interventions may reduce burnout, and system-level approaches have been emphasized for improving clinician wellbeing (30–33, 37). Because residents in professional master’s programs face concurrent clinical and academic demands, attention to research workload, qualification assessment, and career uncertainty may also be relevant (9, 23, 24). These suggestions should be understood as program-level considerations rather than interventions proven effective by the present observational study.

The findings may also be relevant to discussion of pediatric workforce sustainability in China, but national policy implications should be drawn cautiously from this single-center cohort. Pediatricians in China have faced workforce shortages, heavy workload, and retention challenges (1–4). If the observed stage-related difference in emotional exhaustion is replicated across institutions and regions, residency training could represent one potential window for preventive support. However, the present study did not measure professional engagement, specialty identification, intention to leave practice, or workforce retention, and therefore cannot establish downstream workforce consequences. Larger multicenter studies are needed before extrapolating these results to pediatric residents outside professional master’s programs, to other Chinese training systems, or to residency programs in other countries.

This study has several strengths. It used a longitudinal repeated-measures design rather than a cross-sectional comparison of different trainees, allowing assessment of stage-related differences within the same cohort across PGY1–PGY3. The panel was complete, with 50 residents contributing 150 observations, and GEE models accounted for within-person clustering. The study also assessed several related but distinct psychological domains using widely used instruments. Several limitations require emphasis. First, the sample comprised only 50 independent residents from a single tertiary teaching hospital. The 150 repeated observations improve longitudinal characterization but do not substitute for a larger number of independent participants. Statistical power to detect small stage-related differences was therefore limited, and type II error is possible; non-significant results should not be interpreted as evidence that no stage difference exists. External validity is also limited: the findings may not generalize to residents at other institutions or regions of China, residents who are not enrolled in professional master’s programs, or residency systems in other countries. Second, all psychological outcomes were self-reported, and PHQ-9 and burnout cutoffs were used as screening indicators rather than diagnostic definitions. Third, internal consistency was relatively low for DP (*α* = 0.584) and PA (*α* = 0.631). Greater measurement error in these subscales may attenuate associations, widen uncertainty, and reduce power, potentially contributing to non-significant DP and PA findings; these subscale results should therefore be interpreted cautiously.

Fourth, only sex and cohort year were included as covariates. Important time-varying determinants—including weekly working hours, workload, night-shift frequency, rotation intensity, sleep quality, research workload, mentoring, family responsibilities, previous psychiatric history, socioeconomic pressures, and major life events—were unavailable. Residual confounding could therefore bias stage-related associations in either direction, and the observational design does not support causal attribution to training stage. Fifth, the study did not include a pre-residency baseline or a contemporaneous comparison group; training stage cannot be fully separated from elapsed time, aging, calendar-period effects, or other secular changes, and adjustment for cohort year cannot eliminate these influences. Sixth, the exchangeable GEE working correlation was chosen for parsimony, but no sensitivity analysis across alternative correlation structures was performed. Finally, six related continuous outcomes were examined without multiplicity adjustment. The reported *p* values are nominal, the exploratory composite index should not be regarded as an independent confirmatory endpoint, and statistically significant findings require replication in larger prespecified studies. In addition, the exploratory participant-level transition analysis used conventional MBI cut points that are useful for descriptive screening but are not diagnostic thresholds; conditional percentages based on the five PGY1 screen-negative residents are particularly imprecise and should be interpreted cautiously.

In conclusion, this study identified a stage-related difference in emotional exhaustion among pediatric residents enrolled in professional master’s programs, with higher adjusted MBI-EE scores at PGY3 than at PGY1. The exploratory composite burnout index showed a parallel pattern, whereas adjusted differences in depersonalization, personal accomplishment, perceived stress, and depressive symptoms were not statistically significant. These findings are hypothesis-generating rather than evidence of a causal training-stage effect and should be interpreted in light of the small single-center sample, residual confounding, measurement limitations, and multiple testing. Replication in larger multicenter cohorts with detailed time-varying occupational measures is needed. Meanwhile, repeated monitoring and supportive training environments may be reasonable considerations for residency programs concerned with trainee wellbeing.

## Data Availability

The raw data supporting the conclusions of this article will be made available by the authors, without undue reservation.
