# Supplementary material for: Longitudinal trajectories of burnout, perceived stress, and depressive symptoms among pediatric residents enrolled in professional master’s programs in China: a repeated-measures cohort study
Source: Front Public Health. 2026 Sep 15;14:1923629. doi: 10.3389/fpubh.2026.1923629 (PMC13619912; doi:10.3389/fpubh.2026.1923629)
Supplement: Supplementary file 1 [file Table_1.DOCX]

**Supplementary Table S1. Severity distribution of stress, depressive symptoms, and burnout indicators**

| **Domain / indicator** | **Category / threshold** | **Overall, n (%)** | **PGY1, n (%)** | **PGY2, n (%)** | **PGY3, n (%)** |
| --- | --- | --- | --- | --- | --- |
| **PSS-10 severity distribution** | | | | | |
| PSS-10 | Low (0–13) | 19 (12.7) | 9 (18.0) | 6 (12.0) | 4 (8.0) |
| PSS-10 | Moderate (14–26) | 104 (69.3) | 32 (64.0) | 38 (76.0) | 34 (68.0) |
| PSS-10 | High (27–40) | 27 (18.0) | 9 (18.0) | 6 (12.0) | 12 (24.0) |
| **PHQ-9 severity distribution** | | | | | |
| PHQ-9 | None/minimal (0–4) | 1 (0.7) | 0 (0.0) | 0 (0.0) | 1 (2.0) |
| PHQ-9 | Mild (5–9) | 29 (19.3) | 14 (28.0) | 9 (18.0) | 6 (12.0) |
| PHQ-9 | Moderate (10–14) | 54 (36.0) | 17 (34.0) | 22 (44.0) | 15 (30.0) |
| PHQ-9 | Moderately severe (15–19) | 43 (28.7) | 10 (20.0) | 15 (30.0) | 18 (36.0) |
| PHQ-9 | Severe (20–27) | 23 (15.3) | 9 (18.0) | 4 (8.0) | 10 (20.0) |
| **Burnout and depressive-symptom indicators** | | | | | |
| Burnout screening positive | EE ≥ 27 or DP ≥ 10 | 141 (94.0) | 45 (90.0) | 48 (96.0) | 48 (96.0) |
| High burnout by all three dimensions | EE ≥ 27, DP ≥ 10, and PA ≤ 33 | 82 (54.7) | 21 (42.0) | 28 (56.0) | 33 (66.0) |
| PHQ-9 screening positivity | PHQ-9 ≥ 10 | 120 (80.0) | 36 (72.0) | 41 (82.0) | 43 (86.0) |
| Moderately severe or severe depressive symptoms | PHQ-9 ≥ 15 | 66 (44.0) | 19 (38.0) | 19 (38.0) | 28 (56.0) |

**Note.** Percentages were calculated using all repeated observations for the overall column (N = 150) and within each training stage for PGY-specific columns (n = 50 per stage). PSS-10 categories were low (0–13), moderate (14–26), and high (27–40). Burnout screening positive was defined as EE ≥ 27 or DP ≥ 10; high burnout by all three dimensions was defined as EE ≥ 27, DP ≥ 10, and PA ≤ 33. PHQ-9 categories and thresholds are symptom-screening classifications and do not establish a clinical diagnosis. PGY = postgraduate year; PSS-10 = 10-item Perceived Stress Scale; PHQ-9 = Patient Health Questionnaire-9; EE = emotional exhaustion; DP = depersonalization; PA = personal accomplishment.
