# Supplementary material for: Longitudinal trajectories of burnout, perceived stress, and depressive symptoms among pediatric residents enrolled in professional master’s programs in China: a repeated-measures cohort study
Source: Front Public Health. 2026 Sep 15;14:1923629. doi: 10.3389/fpubh.2026.1923629 (PMC13619912; doi:10.3389/fpubh.2026.1923629)
Supplement: Supplementary file 2 [file Table_2.DOCX]

**Supplementary Table S2. Internal consistency reliability of the study scales**

| **Scale** | **Items, n** | **Cronbach's alpha** | **Item range** | **Note** |
| --- | --- | --- | --- | --- |
| Full 22-item MBI-HSS item set | 22 | 0.843 | 0–6 per item | Reported descriptively; not interpreted as a unidimensional total scale. |
| MBI-EE | 9 | 0.734 | 0–6 per item | Emotional exhaustion subscale |
| MBI-DP | 5 | 0.584 | 0–6 per item | Depersonalization subscale |
| MBI-PA | 8 | 0.631 | 0–6 per item | Personal accomplishment subscale |
| PSS-10 | 10 | 0.814 | 0–4 per item | Items 4, 5, 7, and 8 were reverse-scored before calculating the total score. |
| PHQ-9 | 9 | 0.836 | 0–3 per item | Depressive symptoms scale |

**Note.** Cronbach's alpha values were calculated using all 150 repeated observations for descriptive reliability assessment. The lower alpha values for DP and PA indicate limited internal consistency and should be considered when interpreting estimates for these subscales. MBI-HSS = Maslach Burnout Inventory–Human Services Survey; EE = emotional exhaustion; DP = depersonalization; PA = personal accomplishment; PSS-10 = 10-item Perceived Stress Scale; PHQ-9 = Patient Health Questionnaire-9.
