# Supplementary material for: Longitudinal trajectories of burnout, perceived stress, and depressive symptoms among pediatric residents enrolled in professional master’s programs in China: a repeated-measures cohort study
Source: Front Public Health. 2026 Sep 15;14:1923629. doi: 10.3389/fpubh.2026.1923629 (PMC13619912; doi:10.3389/fpubh.2026.1923629)
Supplement: Supplementary file 3 [file Table_3.DOCX]

**Supplementary Table S3. Participant-level longitudinal burnout status patterns across PGY1–PGY3**

*Definitions. Screening positive was defined as EE ≥ 27 or DP ≥ 10; three-dimension high burnout was defined as EE ≥ 27, DP ≥ 10, and PA ≤ 33. These cut points are screening classifications, not diagnostic thresholds.*

**A. Complete three-stage status sequences**

| **PGY1** | **PGY2** | **PGY3** | **n** | **% of cohort** |
| --- | --- | --- | --- | --- |
| **Screening-positive definition** |  |  |  |  |
| Positive | Positive | Positive | 42 | 84.0 |
| Positive | Positive | Negative | 1 | 2.0 |
| Positive | Negative | Positive | 1 | 2.0 |
| Positive | Negative | Negative | 1 | 2.0 |
| Negative | Positive | Positive | 5 | 10.0 |
| **Three-dimension high-burnout definition** |  |  |  |  |
| High | High | High | 8 | 16.0 |
| High | High | Not high | 2 | 4.0 |
| High | Not high | High | 6 | 12.0 |
| High | Not high | Not high | 5 | 10.0 |
| Not high | High | High | 13 | 26.0 |
| Not high | High | Not high | 5 | 10.0 |
| Not high | Not high | High | 6 | 12.0 |
| Not high | Not high | Not high | 5 | 10.0 |

*Note. Patterns with n = 0 are omitted. Percentages use the full cohort (N = 50) as the denominator and sum to 100% within each definition.*

**B. Persistent and PGY1-to-PGY3 transition summaries**

| **Definition** | **Positive at all 3 stages** | **PGY1 positive → PGY3 negative** | **PGY1 negative → PGY3 positive** | **Negative at all 3 stages** | **PGY1 positive** | **PGY2 positive** | **PGY3 positive** |
| --- | --- | --- | --- | --- | --- | --- | --- |
| Screening positive | 42/50 (84.0%) | 2/45 (4.4%); 2/50 (4.0%) | 5/5 (100.0%); 5/50 (10.0%) | 0/50 (0.0%) | 45/50 (90.0%) | 48/50 (96.0%) | 48/50 (96.0%) |
| Three-dimension high burnout | 8/50 (16.0%) | 7/21 (33.3%); 7/50 (14.0%) | 19/29 (65.5%); 19/50 (38.0%) | 5/50 (10.0%) | 21/50 (42.0%) | 28/50 (56.0%) | 33/50 (66.0%) |

*Note. For PGY1-to-PGY3 transitions, the first percentage uses the applicable baseline-status subgroup and the second uses the full cohort. No inferential testing was performed. Screening positivity was more persistent than the stricter three-dimension classification. Because only five residents were screen-negative at PGY1, that conditional percentage is imprecise and should be read with the count and full-cohort percentage.*
