# Supplementary material for: Longitudinal trajectories of burnout, perceived stress, and depressive symptoms among pediatric residents enrolled in professional master’s programs in China: a repeated-measures cohort study
Source: Front Public Health. 2026 Sep 15;14:1923629. doi: 10.3389/fpubh.2026.1923629 (PMC13619912; doi:10.3389/fpubh.2026.1923629)
Supplement: Supplementary file 4 [file Table_4.DOCX]

**Supplementary Table S4. Contextual comparison of burnout across medical training and pediatric practice**

| **Career stage** | **Study and setting** | **Instrument / definition** | **Domain findings** | **Overall burnout finding** | **Contextual interpretation** |
| --- | --- | --- | --- | --- | --- |
| **Present longitudinal cohort** |  |  |  |  |  |
| Current study: PGY1 | China; pediatric professional master’s residents; n=50 | Full 22-item MBI-HSS | EE 26.50 ± 6.22; DP 15.20 ± 4.15; PA 23.92 ± 5.87 | Screen positive 90.0%; all-three high 42.0% | Same cohort followed annually |
| Current study: PGY2 | Same participants; n=50 | Full 22-item MBI-HSS | EE 27.22 ± 6.71; DP 15.76 ± 3.57; PA 23.90 ± 5.73 | Screen positive 96.0%; all-three high 56.0% | Within-participant second assessment |
| Current study: PGY3 | Same participants; n=50 | Full 22-item MBI-HSS | EE 29.24 ± 5.87; DP 16.44 ± 3.91; PA 24.98 ± 4.79 | Screen positive 96.0%; all-three high 66.0% | Within-participant third assessment |
| **Published comparators** |  |  |  |  |  |
| Medical students | United States national survey; Dyrbye et al. (33) | Two validated single items adapted from MBI EE and DP domains | Subscale means not reported | Burnout 55.9% | Earlier stage; not full MBI-HSS |
| Residents/fellows | United States national survey; Dyrbye et al. (33) | Same two MBI-derived items | Subscale means not reported | Burnout 60.3% | Training-stage peak in that survey |
| Early-career physicians | United States national survey; Dyrbye et al. (33) | Same two MBI-derived items | Subscale means not reported | Burnout 51.4% | ≤5 years in practice |
| Pediatric residents | United States multisite samples; Kemper et al. (34) | Full MBI in 2016 and 2017 | Subscale means not reported | Burnout 56% (2016), 54% (2017) | Large national pediatric resident samples |
| Pediatric residents | Saudi Pediatrics Residency Program, Riyadh; n=264; Bin Dahmash et al. (35) | Full MBI-HSS; conventional domain cut points | High EE 63.6%; high DP 27.7%; low PA 48.5% | All-three high 15.9% | Cross-sectional pediatric residency comparator |
| Medical professionals | Six provinces in China; n=25,120; Xiao et al. (36) | Full MBI-HSS | High EE 35.5%; high DP 30.0%; low PA 38.4% | EE-high or DP-high 60.8%; all-three 11.2% | Mixed professions; shorter work experience and pediatrics associated with greater risk |
| Residents + staff pediatricians | Argentine pediatric hospital; 39 residents + 68 staff pediatricians; Pistelli et al. (37) | Full 22-item MBI | EE 30.60 ± 10.98; DP 10.51 ± 5.95; PA 35.76 ± 7.32 | Overall classification not presented in directly comparable form | Longer practice associated with lower DP and higher PA |

*Note. The MBI form, sampling frame, setting, calendar period, and burnout definition differed across studies. Pre-residency data sometimes used validated MBI-derived single items rather than the full MBI-HSS. Values are provided for descriptive context only and should not be interpreted as a common benchmark or pooled comparison. EE = emotional exhaustion; DP = depersonalization; PA = personal accomplishment; MBI-HSS = Maslach Burnout Inventory–Human Services Survey; PGY = postgraduate year.*

**References**

33. Dyrbye LN, West CP, Satele D, Boone S, Tan L, Sloan J, et al. Burnout among U.S. medical students, residents, and early career physicians relative to the general U.S. population. Acad Med. (2014) 89:443–51. doi: 10.1097/ACM.0000000000000134

34. Kemper KJ, Schwartz A, Wilson PM, Mahan JD, Schubert CJ, Staples BB, et al. Burnout in pediatric residents: three years of national survey data. Pediatrics. (2020) 145:e20191030. doi: 10.1542/peds.2019-1030

35. Bin Dahmash A, Alajmi MF, Aldayel AY, Alotaibi YT, Altoum SM, Alzayed A, et al. Burnout and associated risk factors in pediatric residents. Ochsner J. (2021) 21:152–7. doi: 10.31486/toj.20.0037

36. Xiao Y, Dong D, Zhang H, Chen P, Li X, Tian Z, et al. Burnout and well-being among medical professionals in China: a national cross-sectional study. Front Public Health. (2022) 9:761706. doi: 10.3389/fpubh.2021.761706

37. Pistelli Y, Perochena J, Moscoloni N, Tarrés MC. Burnout syndrome among pediatricians: bivariate and multivariate analysis. Arch Argent Pediatr. (2011) 109:129–34.
